# Bacteriophage-Resistant *Staphylococcus aureus* Mutant Confers Broad Immunity against Staphylococcal Infection in Mice

**DOI:** 10.1371/journal.pone.0011720

**Published:** 2010-07-22

**Authors:** Rosanna Capparelli, Nunzia Nocerino, Rosa Lanzetta, Alba Silipo, Angela Amoresano, Chiara Giangrande, Karsten Becker, Giuseppe Blaiotta, Antonio Evidente, Alessio Cimmino, Marco Iannaccone, Marianna Parlato, Chiara Medaglia, Sante Roperto, Franco Roperto, Luigi Ramunno, Domenico Iannelli

**Affiliations:** 1 Faculty of Biotechnology, University of Naples, Portici, Naples, Italy; 2 Department of Organic Chemistry and Biochemistry, University of Naples, Naples, Italy; 3 Universitätsklinikum Münster Institut für Medizinische Mikrobiologie, Münster, Germany; 4 School of Agriculture, University of Naples, Portici, Naples, Italy; 5 Department of Pathology and Animal Health, University of Naples, Naples, Italy; National Institute of Allergy and Infectious Diseases, National Institutes of Health, United States of America

## Abstract

In the presence of a bacteriophage (a bacteria-attacking virus) resistance is clearly beneficial to the bacteria. As expected in such conditions, resistant bacteria emerge rapidly. However, in the absence of the phage, resistant bacteria often display reduced fitness, compared to their sensitive counterparts. The present study explored the fitness cost associated with phage-resistance as an opportunity to isolate an attenuated strain of *S. aureus*. The phage-resistant strain A172 was isolated from the phage-sensitive strain A170 in the presence of the M^Sa^ phage. Acquisition of phage-resistance altered several properties of A172, causing reduced growth rate, under-expression of numerous genes and production of capsular polysaccharide. *In vivo*, A172 modulated the transcription of the TNF-α, IFN-γ and Il-1β genes and, given intramuscularly, protected mice from a lethal dose of A170 (18/20). The heat-killed vaccine also afforded protection from heterologous methicillin-resistant *S. aureus* (MRSA) (8/10 mice) or vancomycin-intermediate *S. aureus* (VISA) (9/10 mice). The same vaccine was also effective when administered as an aerosol. Anti-A172 mouse antibodies, in the dose of 10 µl/mouse, protected the animals (10/10, in two independent experiments) from a lethal dose of A170. Consisting predominantly of the sugars glucose and galactose, the capsular polysaccharide of A172, given in the dose of 25 µg/mouse, also protected the mice (20/20) from a lethal dose of A170. The above results demonstrate that selection for phage-resistance can facilitate bacterial vaccine preparation.

## Introduction

*Staphylococcus aureus* can cause minor infections as well as life-threatening diseases. Endocarditis, osteomyelitis, pneumonia, surgical wound and intravascular device infections caused by *S. aureus* represent a major public health concern [1]–[3]. In the United States, about 60% of nosocomial *S. aureus* bloodstream infections and 40–60% of surgical wound infections are caused by methicillin-resistant strains of *S. aureus* (MRSA) [3]. One of these MRSA strains has been reported to cause an alarming number of necrotizing fasciitis cases [3]. Strains of *S. aureus* with reduced susceptibility to vancomycin are also emerging [2], [4]. In the wake of the rising antimicrobial resistance [5], new strategies for the control of *S. aureus* infections are needed. This study describes the development of a vaccine active against *S. aureus*.

In the presence of an antibiotic or a bacteriophage (a bacteria-attacking virus), resistance is clearly beneficial to the bacteria. As might be expected, in such conditions, resistant bacteria are swift to emerge [6]. However, in vivo and in vitro experiments demonstrate that, in the absence of the antibiotic or phage, resistant bacteria often display reduced fitness compared to their sensitive counterparts [6]–[8]. This fitness cost is particularly high in the case of phage-resistant bacterial strains. To infect bacteria, phages often select an essential component of the bacterial cell wall as a receptor [8]. To gain resistance, bacteria must dispose of this component or alter its conformation [8]. However, this strategy is costly to bacteria, which often become less virulent or avirulent. The present study explored the cost of phage resistance to isolate an attenuated strain of *S. aureus*. In a mouse model of infection, this phage-resistant strain prevented infections from diverse and clinically relevant *S. aureus* strains.

## Materials and Methods

### Bacteria

*S. aureus* strains A170, A177, A179 and A181 were isolated from clinical samples collected from patients with infected surgical wounds (one of the patients had diabetes; one wounds from a car incident; the remaining two were patients who underwent abdominal surgery). Patients were hospitalized at the Medical School of the University of Naples Federico II. Clinical samples were streaked on trypticase soy agar (TSA) (Oxoid, Milan, Italy) and single colonies expanded in trypticase soy broth (TSB) (Oxoid). The strains listed above and their phage-resistant mutants (A172, A178, A180, A182) were all identified as *S. aureus* [9]. For in vivo and in vitro experiments, bacteria were grown in TSB at 37°C, harvested while in exponential growth phase (OD_600_: 1.5 to 1.8), centrifuged (8×10^3^×*g* for 10 min) and washed with saline (0.15 M NaCl). Serial 10-fold dilutions in saline were then plated on TSA.

### Mice

Experiments were carried out on female Balb/c mice (aged 8 to 10 weeks) at the animal facility of the University of Naples. Mice were infected via the intramuscular or aerosol routes. In the case of the intramuscular infection, mice received 5×10^6^–10^8^ bacteria (as detailed in each experiment) suspended in sterile saline (100 µl/mouse). In the case of aerosol infection, mice were placed in a nebulizing chamber and exposed to an aerosol solution (10^7^ CFU/ml) for 10 min. When inspected on the day of infection, the lungs displayed about 1.6×10^5^±5.5×10^3^ CFU/g/mouse (average of 3 mice). Organs were dissected and weighed. Samples were homogenized in 1 ml saline and serially diluted in saline. Colony forming units (CFU) were evaluated by the plate count assay and expressed as CFU/g. Animal experiments were approved by the Animal Care Committee of the University of Naples (permit number 86/609/EEC).

### Isolation of the phage-resistant strains

Phage M^Sa^ was isolated from the *S. aureus* strain A170 following induction with mitomycin [10]. Phages M^Sa1^, M^Sa2^ and M^Sa3^ were isolated from *S. aureus* strains A177, A179 and A181, respectively; phage release was again induced with mitomycin [10]. Phage-resistant *S. aureus* strains A172, A178, A180 and A182 were isolated by plating dilutions of overnight susceptible cultures on TSA containing increasing concentrations of the phage used for selection. Ten single colonies growing at the highest phage concentration were selected and subcultured twice on TSA agar in the absence of phage. To ensure stability of resistance, two colonies from each phage-resistant strain were further subcultured (20 times or more) in the absence of phage. Induction of the phage-resistant strains (including A172) with mitomycin excluded the presence of prophages in these strains.

### Titration of anti-A172 antibodies

Mice were immunized with the A172 strain (10^8^ CFU/mouse) and two weeks later sacrificed and the blood pooled. The protein A gene is under-expressed in the A172 strain. Yet, to avoid interference with the protein A possibly present on the bacterial surface, the wells of a 96-well plate (Falcon, Milan) were coated with the *S. aureus* protein A negative strain Wood 46 [11], quenched with 3% bovine serum albumin (50 µl/well; 2 h), washed with PBS, incubated with anti-A172 serum diluted (10^−2^–10^−4^) in PBS (50 µl/well; 2 h), washed with PBS and incubated, in succession, with peroxidase-labelled rat anti mouse IgG (50 µl/well; 2h) and peroxidase substrate (100 µl/well; Bio-Rad, Milan).

### Carbohydrate analysis

Teichoic acids from the A170 (A170^TA^) and A172 (A172^TA^) isolates were prepared as described [12] and analyzed gas chromatography-combined mass spectrometry. Monosaccharides were identified as acetylated O-methyl glycosides derivatives, fatty acids or O-methyl ester derivatives. After methanolysis with methanolic HCl (2M HCl/CH_3_OH; 85°C, 24 h) and acetylation with acetic anhydride in pyridine (85°C; 20 min), samples were analyzed by gas chromatography-combined mass spectrometry (GC-MS) and compared with standards. GC-MS analyses were carried out on a Hewlett-Packard 5890 instrument using SPB-5 capillary column (0.25×3 m; Supelco, PA). Ring size and attachment points were determined by methylation analysis as described [13]. The sample (1–2 mg) was methylated with CH_3_I/NaOH in DMSO, hydrolysed with 2M trifluoroacetic acid (100°C, for 4 h), reduced with NaBD_4_, acetylated with acetic anhydride in pyridine and analyzed by GC-MS. The temperature programme was: 150°C for 5 min and then 5°C/min to 300°C over 10 min.

### Capsular polysaccharide purification

The A172 strain (100 ml) was grown in TSB. When in the exponential growth phase, the culture was centrifuged (8×10^6^×g for 10 min) and the supernatant precipitated with ethanol (four volumes) in the cold (−20C overnight). Supernatant and precipitate were separated by centrifugation (8×10^6^×g for 10 min). The precipitate, insoluble in H2O and several organic solvents (methanol, ethanol, chloroform, or dimethyl sulfoxide), was discarded. The supernatant was dialyzed (cut-off of the dialysis tube: 3500 Daltons) against water (3 days), lyophilised, weighed and tested for the capacity to protect mice. Mice vaccinated with the supernatant (25 µg/mouse) were fully protected against a lethal dose of A170 bacteria. This biologically active fraction (3.4 g) was hydrolysed with 0.1 M HCl at 100°C for 48 h. The mixture was neutralized with 2 M NaOH and reduced with sodium borohydride in distilled water (3 g/0.5 ml). The alditols were acetylated with pyridine (100 µl) and acetic anhydride (100 µl) at 85°C for 30 min. The monoses were identified as acetate alditols by gas liquid chromatography (GLC) [14] using the Agilent GC-MS 7890A instrument (Waghaeusel-Wiesental, Germany) equipped with a capillary column (30 m×0.25 mm, 0.25 µm film thickness) of SPB-5 (Sigma Aldrich, Milan, Italy) and applying a temperature gradient of 150°C (3 min) to 320°C at 3°C/min.

### Detection of A172 revertants

Individual wells of 96-well plates were filled with 200 µl TSB medium and inoculated with one colony of A172 bacteria isolated from mice vaccinated with the same strain and then grown in vitro for 15 generations under stress conditions (10 µg/ml novobiocin) [15]. Bacteria were grown for 3 h at 37°C. Individual cultures (10^9^ CFU/200 µl) were quantitatively spread over a phage lawn (10^12^ PFU/5 ml soft agar). Bacteria were thus surrounded by large numbers of virus particles. Plates were inspected for the presence of plaques of lysis during the next 4 days. The detection limit of the test was established by carrying out the test on the same A172 cultures spiked with 12, 6, 3 or 1 A170 M^Sa^-sensitive bacteria. The dilutions with 12, 6, 3 or 1 A170 bacteria were assayed by plate count in triplicate.

The presence of revertants was also investigated by an independent method, based on the increase of phage titre of a liquid culture upon addition of phage-sensitive bacteria [16]. A172 bacteria grown in the presence of novobiocin (10^9^ CFU/200 µl) were incubated for 1 h with the M^SA^ phage (10^5^ PFU). The cultures were then transferred quantitatively on a lawn of the A170 M^Sa^-sensitive bacteria (10^5^ CFU/4 ml soft agar) to determine whether the original phage titre increased. The detection limit of the test was established by testing in parallel the phage titre increase that occurred in the same A172 cultures (10^9^ CFU/200 µl) spiked with 12, 6, 3 or 1 A170 M^Sa^-sensitive bacteria. The dilutions with 12, 6, 3 or 1 A170 bacteria were assayed as described above.

### Phage lysis inhibition

A170 or A172 bacteria (10^5^ CFU/100 µl) were grown (4 h) in 2 ml TSB, in the presence or absence of: 5–20 mM N-acetyl-glucosamine (GlcNAc), 5–20 mM glucose (Sigma, Milan), 0.1–4 nmol/tube teichoic acid from A170 (A170^TA^) or A172 (A172^TA^). The M^Sa^ phage (10^9^ PFU7tube) was then added and bacterial growth (OD_600_) measured 30 min later.

### Phage inactivation by N-acetyl-glucosaminidase

A170 or A172 bacteria (10^5^ CFU/100 µl) were grown (4 h) in 2 ml TSB, in the presence or absence of N-acetyl-glucosaminidase from *Canavalia ensiformis* (Sigma; 4 U/tube). The M^Sa^ phage was then added (10^9^ PFU) and bacterial growth (OD_600_) measured 30 min later.

### Pulsed-field electrophoresis of *S. aureus* strains

The procedure adopted was that described in [17]. Briefly, inserts of intact DNA were digested in 200 µl of appropriate buffer supplemented with 40 U of *Sma* I (Promega, Milan). Pulsed field gel electrophoresis (PFGE) of the restriction digests was performed by using the CHEF system (Bio-Rad Laboratories, Hercules, CA, USA) with 1% (wt/vol) agarose gels and 0.5×TBE as running buffer, at 10°C. Restriction fragments were resolved in a single run, at constant voltage of 6 V cm^2^ and an orientation angle of 120° between electric fields, by a single phase procedure for 24 h with a pulse ramping between 1 and 50s.

### Other methods

The transcription level of bacterial virulence genes and mouse cytokine genes was analyzed by real-time reverse transcription PCR (RT-PCR) as described [18]. Fluorescein isothiocyanate labelled A170 (A170^FITC^) and A172 (A172^FITC^) bacteria were prepared as described [19]. Troponin was measured using the Mini Vidas automated immunoassay analyzer and the Vidas troponin kit (bioMereux, Florence, Italy). The sequence type of the *S. aureus* strains were determined by sequencing the hypervariable region of *S. aureus* protein A gene (*spa*) as described [20]. The *spa* types were assigned using the Ridom *spa*-server (http://spaserver.ridom.de). Multilocus sequence typing (MLST) of *S. aureus* strains was carried out as described [21]. The bacterial genes *eta*, *etb*, *tst*, *lukS-PV–lukF-PV*, *lukE-lukD* and *lukM*, (coding for the exfoliative toxin A, exfoliative toxin B, toxic shock syndrome toxin1, Panton-Valentine leukocidin components S and F, the leukotoxin LukELukD, and the leukotoxin LukM, respectively) were detected by PCR [10]. The staphylococcal enterotoxin (*se*) genes *se-a*, *se-b, se-c*, se-*d*, and *se-e* were detected as described [22]. The restriction endonucleases analysis-enterotoxin gene cluster (REA-*egc*) type was carried out as described [23]. Survival rates of mice were analyzed using Fisher's exact test. Bacterial counts and gene expression levels were analyzed using the paired t test (P values are two-tailed values). The molar concentration of the teichoic acids from the *S. aureus* strains A170 and A172 were calculated attributing to A170 and A172 the molecular weight of 6350 daltons (the average molecular weight of the teichoic acid from *S. aureus* strain Copenhagen, which ranges from 5400 to 7300 daltons) [24]

## Results

### Isolation and characterization of A172

**Figure 1:**
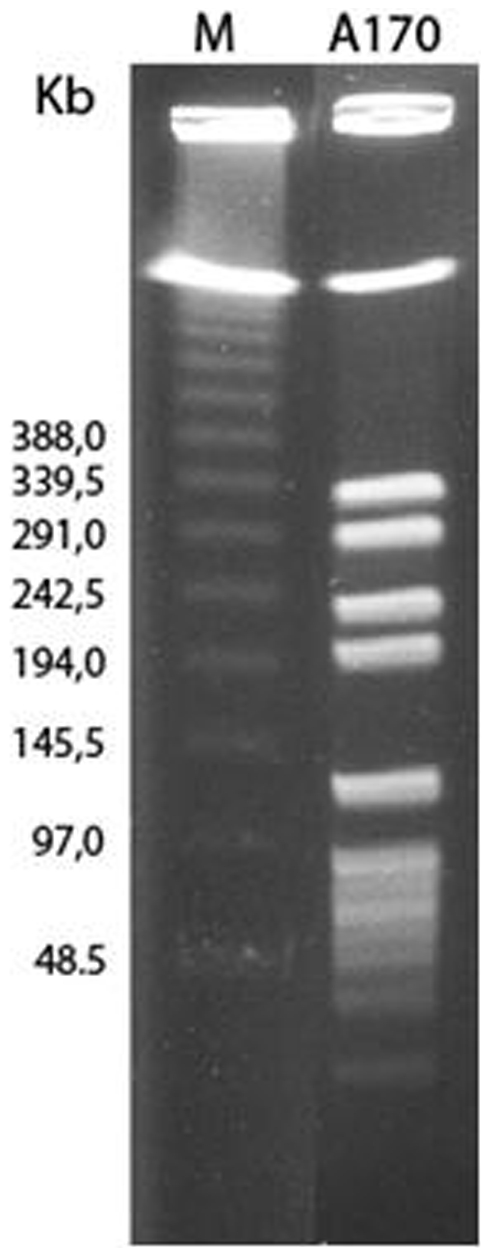
Pulsed field electrophoresis pattern of *Sma I* digests from the *Staphylococcus aureus* A170 strain. The A172 strain (not shown) displayed an identical pattern. M: DNA Size Standard, Lambda Ladder (Concatemers of λ cl857 Sam7) (Bio-Rad Laboratories, Hercules, CA).

The strains A170 and A172 were positive for the exfoliative toxin B, the Panton-Valentine leukocidin, while negative for the exfoliative toxin A, toxic shock syndrome toxin 1, the leukotoxin LukELukD, the leukotoxin LukM and the staphylococcal enterotoxins-a, -b, -c, -d and –e. The strains A170 and A172 displayed also the same egc type (egc-4), the multilocus sequence type (ST 45) and spa-type (t6668) (spa-type repeats succession: 08-16-02-16-34-13-17-34-34-34-34-34, equivalent to Kreiswirth's IDs: XKAKBEMBBBBB). According to the Ridom *spa*-server (http://spaserver.ridom.de), t6668 represents a novel spa type. PFGE analysis displayed a close genetic relation between the parental phage M^Sa^-sensitive strain A170 and the derivative phage-resistant strain A172, as proved by the identical PFGE pattern of the *Sma I* digests (Figure 1). A172 was isolated by growing the parental strain A170 in the presence of increasing concentrations of the M^Sa^ phage [10]. In addition to M^Sa^, the strain A172 is also resistant to phages M^Sa1^, M^Sa2^ and M^Sa3^. Prolonged liquid subculture for several months in the absence of phage or prolonged storage at −80°C did not alter the resistance of A172 to M^Sa^.

The strain was also highly stable in vivo. A172 bacteria were isolated from a vaccinated mouse, grown for 15 generations under stress conditions (10 µg/ml novobiocin) [15] and used to establish 400 independent bacterial cultures, which yielded a total number of about 4×10^11^ bacteria (10^9^, the number of cells/culture×400, the number of cultures). Plated with phage in excess (10^12^ PFU/5 ml soft agar), the 400 independent bacterial cultures did not yield any plaque of lysis. Control cultures (10^9^ CFU A172/200 µl spiked with 3 A170 bacteria), assayed in triplicate in three independent experiments, displayed all at least one plaque and, on average, 2.2±0.7 plaques/culture (number of replicas: 9). The absence of A172 revertants was confirmed in a second independent experiment. When the M^Sa^ phage (10^5^ PFU) was mixed with the A172 bacteria grown under stress conditions (10^9^ CFU/200 µl), no measurable increase in the phage titre occurred. Control cultures (10^9^ CFU A172/200 µl spiked with 3 A170 bacteria), compared to the non-spiked cultures, displayed an increase in phage titre from 1.4×10^5^±24×10^3^ to 1.2×10^6^±0.5×10^3^ (n: 9; P: 0.0057). Conservatively, it is estimated that A172 revertants occur at a frequency <3×10^−9^.

**Figure 2:**
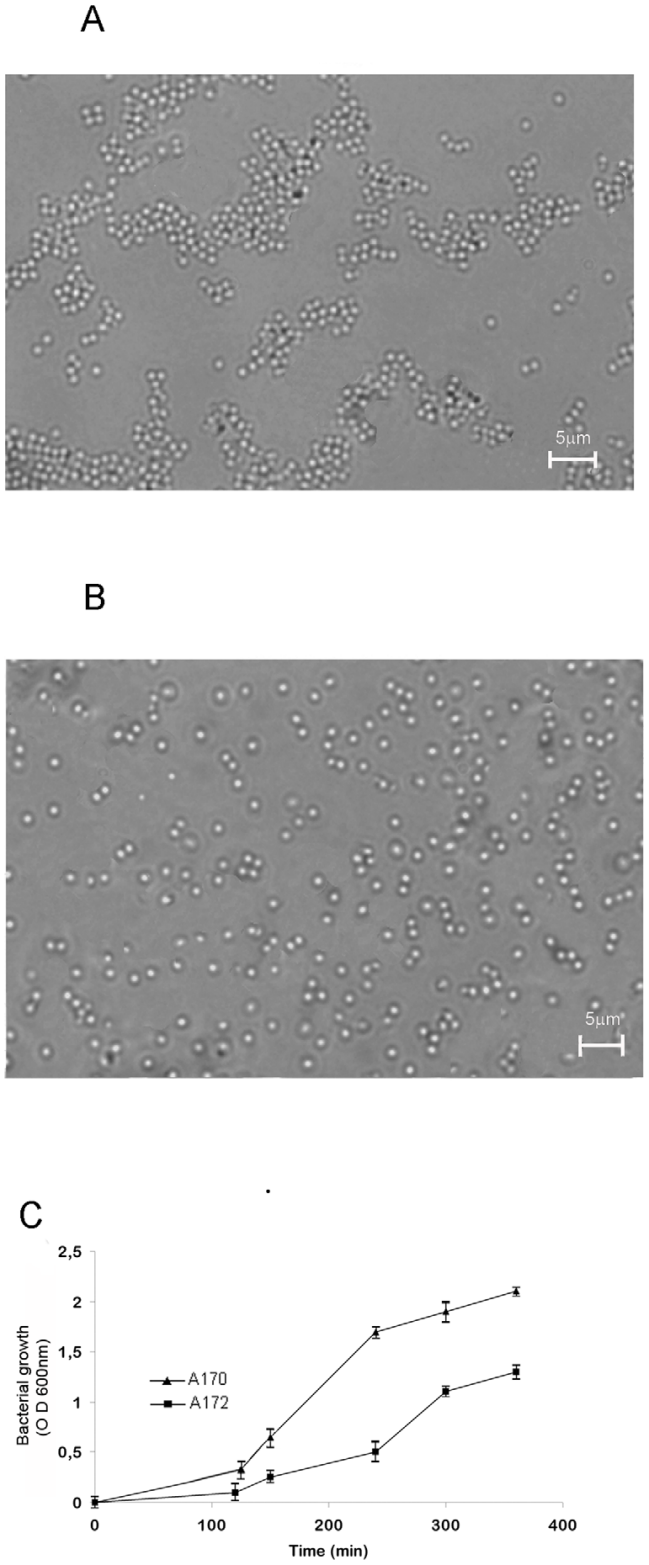
Differences between the phage M^Sa^-sensitive strain A170 and the phage M^Sa^-resistant strain A172. The strain A172 grows in larger clumps (A) compared to the strain A170 (B). Bacteria were grown in liquid culture and collected for microscopic examination at the early stationary phase. (C) The A172 strains displays also a slower growth rate, compared to A170.

**Figure 3:**
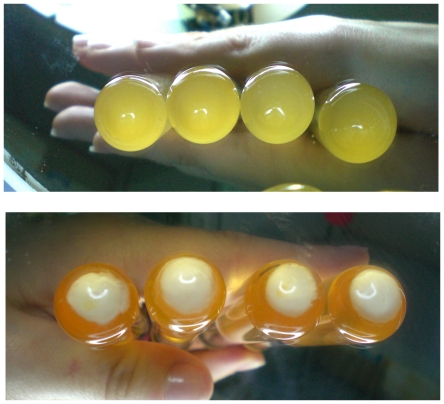
In *S. aureus*, phage-resistance comes with production of capsular polysaccharide. (A) Strains A170, A177, A179, A181 (sensitive to the phages M^SA^, M^SA1^, M^Sa2^, M^SA3^, respectively). (B) Strains A172, A178, A180, A182 (resistant to the phages M^SA^, M^SA1^, M^Sa2^, M^SA3^, respectively).

**Figure 4:**
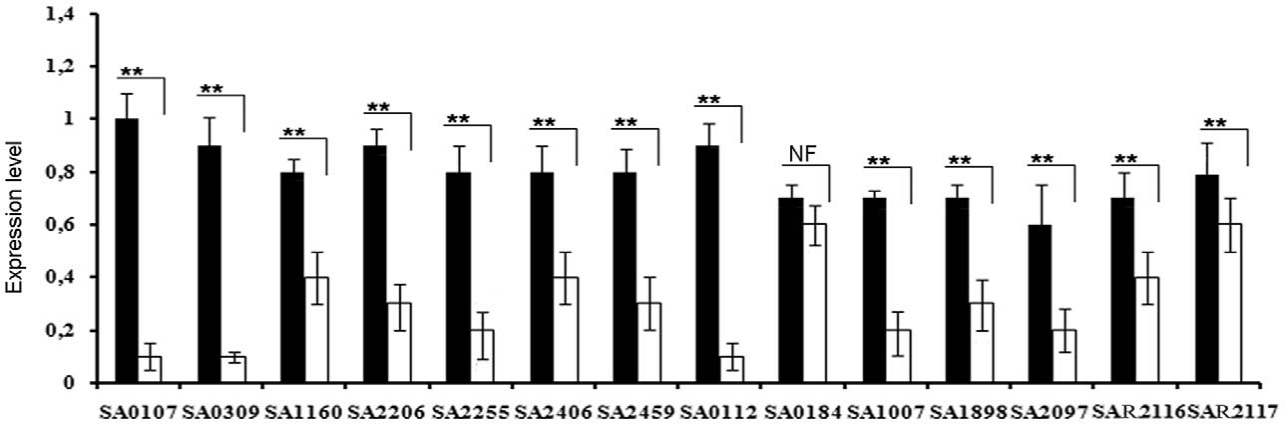
Expression levels of *S. aureus* A170 and A172 virulence factors. Acquisition of phage-resistance by A172 is accompanied by extensive alterations in virulence gene expression levels. Out of the 14 ORFs examined, 13 are significantly under-expressed, compared to the phage-sensitive strain A170. Bacteria were collected for transcriptional analysis during the exponential growth phase. SA0107 (*spa*, IgG binding protein A precursor); SA0112 (hypothetical protein, similar to cysteine synthase); SA0184 (hypothetical protein); SA039 (*geh*; glycerol ester hydrolase); SA1007 (α-haemolysin); SA1160 (*nuc*; thermonuclease); SA1898 (hypothetical protein, similar to SceD precursor); SA2097 (hypothetical protein, similar to secretory antigen precursor SsaA); SA2206 (*sbi*; IgG-binding protein SBI); SA2255 (oligopeptide transporter substrate binding protein); SA2406 (*gbsA*; glycine betaine aldehyde dehydrogenase gbsA); SA2459 (*ica*; N-glycosyltransferase); SAR216 (*groEL*; chaperonin GROEL); SAR2117 (*groES*; co-chaperonin GRES). ORFs numbers correspond to the *S. aureus* N315 genome sequence. Gene designations (when known) and proteins function are shown in parentheses.

In liquid culture, compared to the parental strain, A172 formed colonies with the tendency to clump together starting at the early stationary phase (Figure 2A–B). Bacterial suspensions of A170 and A172 with the same density (OD_600_: 0.6) were assayed by plate count. In three independent experiments, A170 cultures contained about 3 fold as many CFU as the A172 cultures (3.4×10^7^±6.7×10^6^ vs 1.2×10^7^±2.5×10^6^; P 0.003). A172 displayed reduced growth rate during the exponential phase (Figure 2C) and reduced doubling time (A172: 45 min±2.8 min; A170: 30±2.5 min; P 0.002). Also, unlike A170, A172 produced capsular polysaccharide (Figure 3). Phage resistance altered the transcription of several virulence factors. Among 14 ORFs of A172 analyzed, 13 were significantly down-regulated compared to A170 (Figure 4). Down-regulated ORFs comprise 9 genes, which control virulence factors [25].

### The receptor of the M^Sa^ phage is the N-acetylglucosamine

**Figure 5:**
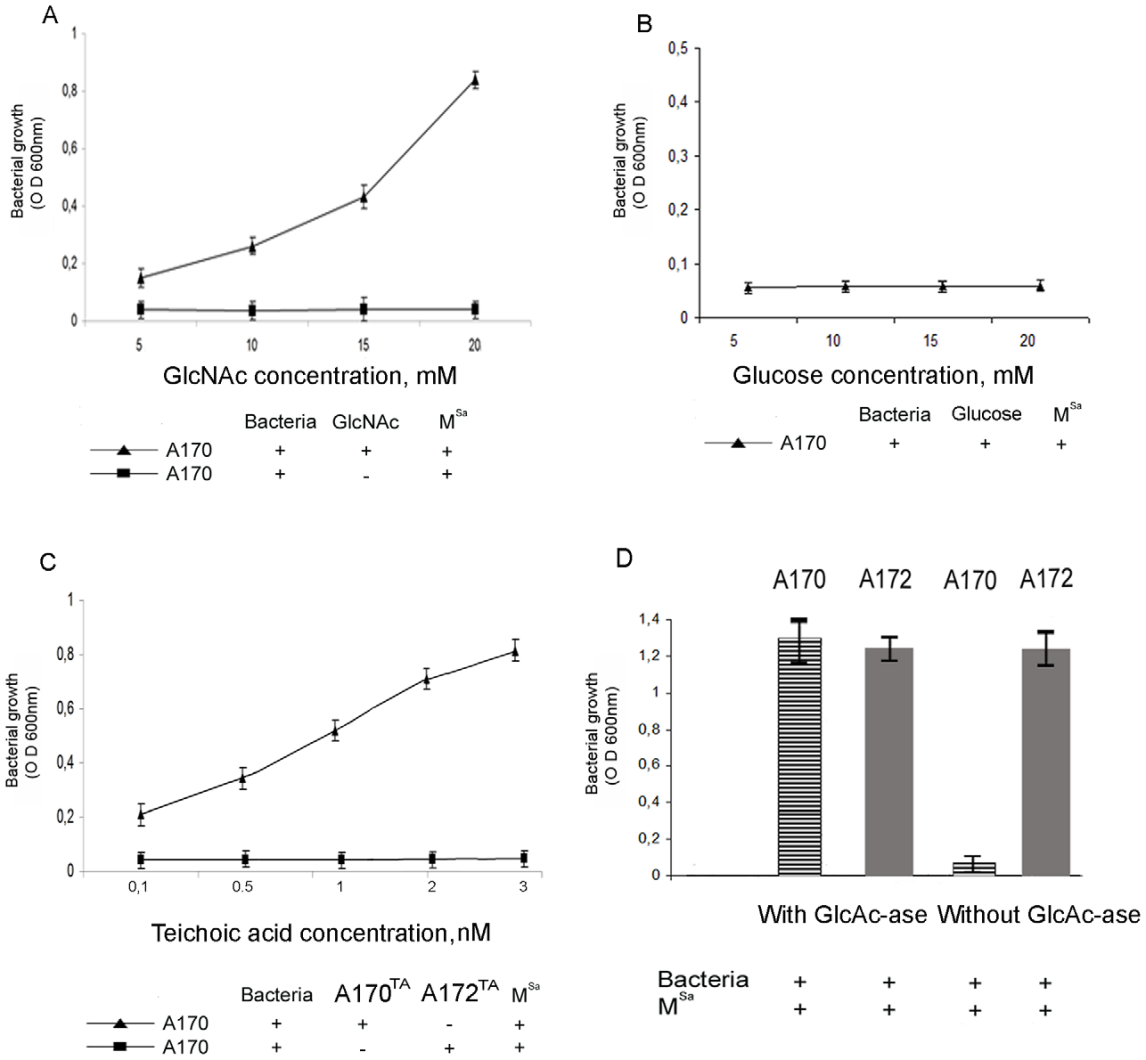
Phage M^SA^ is inhibited by N-acetyl-glucosamine (GlcNAc), the teichoic acid from A170 (A170^TA^) or by treatment with N-acetyl-glucosaminidase (GlcNA-ase) from *Canavalia ensiformis*; phage M^SA^ is not inhibited by the teichoic acid from A172 (A172^TA^) or glucose. (A) GlcNAc inhibits the lysis of the A170 strain by the phage M^Sa^, while glucose (B) does not. (C) A170^TA^ inhibits the lysis of the A170 strain by the phage M^SA^, while A172^TA^ does not. (D) The phage-sensitive strain A170 grows in the presence of the M^Sa^ phage, if pre-treated with GlcAc-ase (4 U/tube; 2 h at 37°C).

**Figure 6:**
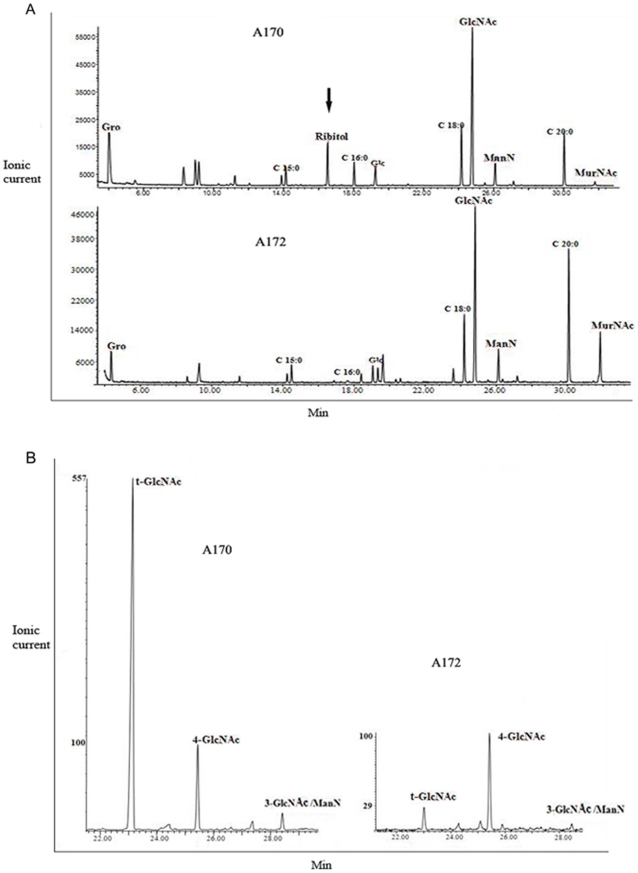
Gas chromatography-combined mass spectrometry spectra of the teichoic acids from the A170 and A172 strains. Acetylated O-methyl glycosides from the phage–sensitive A170 and phage-resistant A172 strains of *S. aureus*. Loss of ribitol (A) and t-GlcNAc linked to ribitol (B) in the teichoic acids is part of the strategy adopted by A172 to gain phage-resistance. The peak of 4-GlcNAc was chosen as reference. Gro: glycerol; GlcNAc: N-acetylglucosamine; ManN: N-acetylmannosamine; MurNAc: N-acetyl muramic acid; Glc: glucose; C15:0: pentadecanoic acid; C16:0: esadecanoic acid; C18:0: octadecanoic acid; C20:0: eicosanoic acid; 4-GlcNAc: 4-linked N-acetylglucosamine; 3GlcAcNMaN: 3-linked N-acetylmannosamine; t-GlcNAc: terminal GlcNAc.

Phages often use teichoic acids for adsorption on the cell wall of Gram positive bacteria. They can use as receptor the glucose side chains of *Bacillus subtilis* teichoic acids [26], [27] or the N-acetylglucosamine (GlcNAc) side chains of *S. aureus* [28]. To identify the receptor site of the M^Sa^ phage, the A170 and A172 strains were grown in the presence or absence of 5–20 mM GlcNAc or 5–20 mM glucose. GlcNAc inhibited the lysis of A170 by phage M^SA^, while glucose did not. GlcNAc inhibition was dose-dependent (Figure 5A–B). The experiment was repeated using the teichoic acid from (A170^TA^) or from (A172^TA^) as inhibitor. Since the quantity of teichoic acid that is isolated from the A170 or A172 strains varies significantly (2.4 mg from 10^10^ CFU A170; 0.9 mg from 10^10^ CFU A172), the experiment was conducted using an ample concentration range of the A170^TA^ and A172^TA^ reagents. Under these conditions, phage inactivation occurred with A170^TA^, but not with A172^TA^ (Figure 5C). A170 bacteria were then treated with N-acetylglucosaminidase (4 U/tube; 2 h at 37°C). Enzymatic hydrolysis of GlcNAc from the A170 bacteria destroyed phage lysis capacity (Figure 5D). Finally, analysis of teichoic acids from the A170 (wild) and A172 (mutant) strains of *S. aureus* displayed the absence of ribitol (Figure 6A) and terminal GlcNAc (t-GlcNAc) residues (Figure 6B) from A172^TA^. Collectively, the experiments described above demonstrate that A172 conforms with the tendency of several bacterial species - *B. subtilis* [26], [27], *S. aureus* [28], *Salmonella enterica* [18], [29] - to acquire phage-resistance by altering a cell wall polysaccharide component.

### The A172 strain prevents *S. aureus* infection

For a more succinct presentation of the results, the distinctive features of the experiments described in this and the following sections are detailed in the Tables. One group of mice was immunized with A172 live (A172-L) and two weeks later infected with a lethal dose of A170. At the time of infection, in the serum of immunized animals the titre of the antibodies against A172-L ranged from 1∶5000 to 1∶12000. Control mice were also infected with a lethal dose of A170. The A172-L vaccine protected 90% of the mice (surviving mice: 18/20) from A170, while the control mice (10/10) died within 4 days (P: 0.008; Table 1, experiment 1). When examined at the end of the experiment (14 days after challenge), the kidneys of surviving A172-L treated animals displayed significantly fewer CFU compared to the kidneys of the control mice at the time of death (Table 1, experiment 1).

**Table 1:** Active protection of A172 in mice challenged with *S. aureus* strain A170.

	Vaccination			Interval vaccination challenge (weeks)	Challenge					
Exp	Dose CFU/mouse	Vaccine	Route		Dose CFU/mouse	Pathogen	Route	Survival	P value^b^	CFU/g^a^ (mean ± SD)
1	10^8^	A172-L^c^	Im^d^	2	10^8^	A170	Im	18/20	0.008	266±11^e^
					-	-	-	0/10		5.6×10^8^±3.5×10^8^
2	5×10^6^	A170	-	2	10^8^	A170	Im	0/10	1	5.7×10^8^±3.2×10^8^
					-	-	-	0/10		5.2×10^8^±1.5×10^8^
3	10^8^	A172-HK^f^	-	2	10^8^	A170	Im	19/20	0.004	260±25^e^
					-	-	-	0/10		3.5×10^8^±1.8×10^8^
4	10^8^	A172-HK	-	20	10^8^	A170	Im	37/40	0.004	200±11^e^
					-	-	-	0/10		5.7×10^8^±3.3×10^8^
5	10^8^	A170-HK	-	2	10^8^	A170	Im	0/10	1	5.2×10^8^±1.8×10^8^
					-	-	-	0/10		4.7×10^8^±2.3×10^8^

a Colony forming units per g of kidneys (experiments 1–4) or lungs (experiment 5); values calculated on 3 mice.

b Two tailed Fisher's exact test.

c A172 Live.

d Intramuscular.

e P<0.001.

f Heat killed A172.

To exclude that the above results represented the response elicited by a sublethal infection with live *S. aureus*, mice were infected with a sublethal dose of A170 and two weeks later challenged with a lethal dose of A170. There was no significant difference in surviving between immunized and control mice (P: 1; Table 1, experiment 2). Thus, the protective response described above is to be ascribed specifically to A172.

Being a live vaccine, A172 poses the risk of reverting to the virulent phenotype. This risk is remote (see the section “Isolation and morphological characterization of A172”), albeit considerable when the use of a large number of vaccine doses is prospected. The study therefore explored whether A172 remained protective once it was heat-killed (15 min at 100°C). Mice were vaccinated with heat-killed vaccine (A172-HK) and 2 weeks later infected with a lethal dose of A170. The vaccine was protective also in this form (P: 0.004; Table 1, experiment 3).

When the interval between vaccination and challenge was extended to 20 weeks, control mice all died within 4 days (10/10); the vaccinated mice (37/40) were alive 14 days after challenge (P: 0.004; Table 1, experiment 4). Thus, the protection provided by the vaccine lasts at least 20 weeks.

One more experiment was designed to ascertain whether also A170 - in the heat-killed form (A170-HK; 15 min at 100°C)- was protective. No significant difference in survival between immunized and control mice was observed (P: 1; Table 2, experiment 5).

**Table 2 pone-0011720-t002:** Protection of heat killed vaccine A172 against drug-resistant *S. aureus* strains.

Vaccination			Interval vaccination challenge (weeks)	Challenge					
Dose (CFU/mouse)	Vaccine	Route		Dose (CFU/mouse)	Pathogen	Route	Survival	P value^b^	CFU/g^a^ (mean ± SD)
10^8^	A172-HK^c^	Im^d^	20	10^8^	A174	Im	8/10	0.02	250±22^e^
				-	-	-	0/10		6.7×10^8^±1.8×10^8^
10^8^	A172-HK	^-^	20	10^8^	A175	Im	9/10	0.01	330±37^e^
				-	-	-	0/10		5.8×10^8^±1×10^8^
10^8^	A172-HK	^-^	20	10^8^	A176	Im	9/10	0.01	380±26^e^
				-	-	-	0/10		5.5×10^8^±3.6×10^8^

a Colony forming units per g of kidneys; values calculated on 3 mice.

b Two tailed Fisher's exact test.

c Heat killed A172.

d Intramuscular.

e P<0.001.

### Additional properties of the A172 vaccine

The efficacy of the A172-HK vaccine was tested against two methicillin-resistant *S. aureus* (MRSA) (A174 and A175) and one vancomycin-intermediate *S. aureus* (VISA) (A176) isolates derived from patients with staphylococcal infections. Three groups of mice were immunized with A172-HK and 20 weeks later infected with a lethal dose of the A174, A175 or A176 isolates. The difference in survival between vaccinated and control mice was statistically significant in each case (P: 0.01–0.02; Table 2).

Patients with cystic fibrosis are highly susceptible to *S. aureus* lung infection [30]. *S. aureus* is also one of the most common causes of pneumonia in paediatric and adult populations [31]. The study therefore explored the efficacy of A172-HK against lung infections. Mice were immunized by aerosol with A172-HK and 20 weeks later infected with A170 by the same route. The vaccine was also effective when administered by aerosol (P: 0.004; Table 3).

**Table 3:** Protection of heat killed vaccine A172 administered by aerosol.

Vaccination			Interval vaccination challenge (weeks)	Challenge					
Dose (CFU/mouse)	Vaccine	Route		Dose (CFU/mouse)	Pathogen	Route	Survival	P value^b^	CFU/g^a^ (mean ± SD)
10^8^	A172-HK^c^	Aer^d^	20	10^8^	A170	Aer	19/20	0.004	216±3^e^
				-	-	-	0/10		7.1×10^8^±3.1×10^8^

a Colony forming units per g of kidneys; values calculated on 3 mice.

b Two tailed Fisher's exact test.

c Heat killed A172.

d Aerosol.

e P<0.001.

Collectively, the experiments described in this and the preceding section demonstrate that A172-HK protects mice against a lethal dose of *S. aureus*; protection correlates with increased survival and reduced colonization lasting at least 20 weeks and extending into drug-resistant isolates of *S. aureus*; the vaccine is effective when administered, in single dose and without adjuvant, by the intramuscular or aerosol routes.

### Anti-A172 antibodies protect against *S. aureus* A170 infection *in vivo*

Two groups of mice (control mice) were injected intramuscularly with normal mouse serum (10 µl or 20 µl diluted 1∶10 with sterile saline, respectively). Two more groups of mice were treated intramuscularly with serum from mice immunized with A172-HK (10 µl or 20 µl diluted 1∶10 with sterile saline, respectively). The titre of the antibodies against A172-HK was 1∶8000. The next day, the mice were all challenged with a lethal dose of *S. aureus* A170. Survival was monitored for seven days. In two independent experiments, the antibodies against A172-HK - given at the dose of 10 µl/mouse - provided protection to all the mice (10/10) (P 0.01;Table 4, experiments 1 and 2). Instead, the same serum - given at the dose of 20 µl/mouse - killed 40%–50% of the mice (P 0.05–0.06; Table 4, experiments 1 and 2). Thus, anti-A172 antibodies can be protective or detrimental, depending upon the dose being given. This phenomenon has been described since the development of serum therapy [32]. In mouse models of *Streptococcus pneumoniae* [32] and *Cryptococcus neoformans* [33], antibody-mediated protection was also observed within a narrow dose range. Since the therapeutic use of antibodies in humans is sometimes associated with myocardial damage [34], [35], mice passively immunized with antibodies against A172-HK were tested for evidence of heart damage by measuring the level of the cardiac troponin T in the serum. This marker is highly specific for myocardial damage [34]. Mice treated with 20 µl antibodies, compared to those treated with 10 µl, displayed a statistically significant higher level of serum troponin T (0.09±0.01 ng/ml vs 0.02±0.01 ng/ml; P: 0.001). It was not investigated whether heart damage was the primary cause of death or a secondary complication of antibody administration. Mice treated with 10 µl or 20 µl antibodies displayed both significantly fewer CFU compared to the mice treated with normal mouse serum (Table 4, experiments 1 and 2).

**Table 4:** Mouse anti A172-HK antibodies protect mice challenged with *S. aureus* A170 strain.

	Passive vaccination			Challenge					
Exp	Treatment	Dose (µl/mouse)	Route	Dose (CFU for mouse)	Strain	Route	Survival	P value^b^	CFU/g^a^ (mean ± SD)
1	Normal mouse serum	10	Im^c^	10^8^	A170	Im	0/10	0.01	5.8×10^8^±1×10^8^ d
	Anti-A172-HK serum	-	-	-	-	-	10/10		90±7
	Normal mouse serum	20	Im	10^8^	A170	Im	0/10	0.05	4.7×10^8^±2×10^7^ d
	Anti-A172-HK serum	-	-	-	-	-	6/10		5.5×10^5^±3.6×10^4^
2	Normal mouse serum	10	Im	10^8^	A170	Im	0/10	0.01	3.5×10^8^±2×10^8^ d
	Anti-A172-HK serum	-	-	-	-	-	10/10		73±6
	Normal mouse serum	20	Im	10^8^	A170	Im	0/10	0.06	6.3×10^8^±1×10^8^ d
	Anti-A172-HK serum	-	^-^	^-^	-	^-^	5/10		2.6×10^5^±10^5^
3	Anti-A172-HK serum absorbed with rat anti-mouse IgG	10	Im	10^8^	A170	Im	0/10		5.4×10^8^±4.2×10^7^

a Colony forming units per g of kidneys; values calculated on 3 mice.

b Two tailed Fisher's exact test.

c Intramuscular.

d P<0.0001.

In an independent experiment, mice were immunized with 10 µl of anti-A172 mouse serum absorbed with rat anti total mouse immunoglobulin fraction (5–10 µl) and diluted to 100 µl with sterile saline. The next day, the mice were challenged with a lethal dose of *S. aureus* A170. Absorption of anti-A172 mouse serum completely eliminated the protective effect of the serum (Table 4, experiments 3). Collectively, the above results demonstrate that the passive protection shown by the antibodies against A172-HK is dose dependent and specific (residing in the antibody fraction of the serum).

### The A172 strain controls inflammation

**Figure 7:**
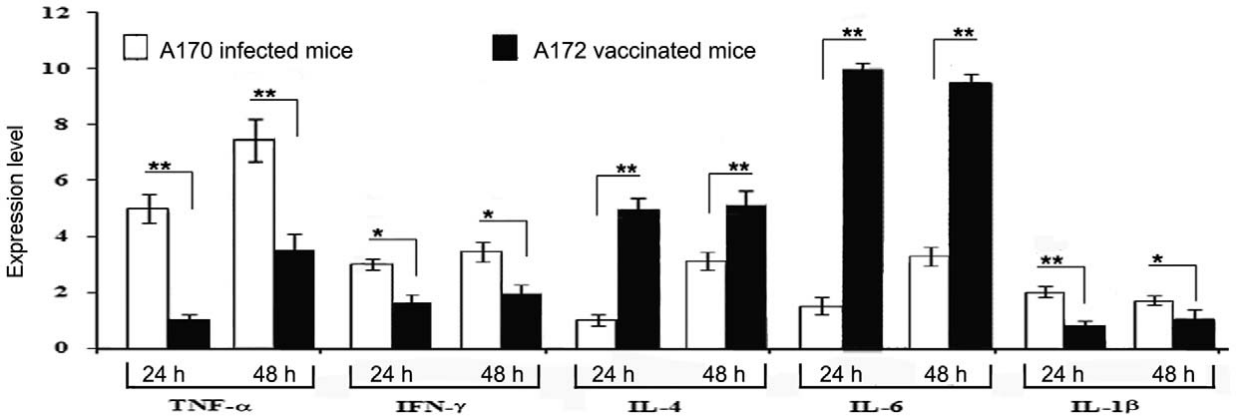
Anti-inflammatory activity of the A172 vaccine. A172 modulates transcription of the genes coding for pro-inflammatory cytokines (TNF-α, INF-γ and IL-1β) and induces transcription of the genes coding for anti-inflammatory cytokines (Il-4 and Il-6).

Ten mice were immunized intramuscularly with A172-HK (10^8^ CFU/mouse). A172 modulated transcription of the genes coding for the pro-inflammatory cytokines TNF-α, IFN-γ and IL-1β, while inducing that of the genes coding for the anti-inflammatory cytokines Il-4 and Il-6, when these genes were analyzed by RT-PCR, 24 and 48 h after immunization (Figure 7). The same pattern of cytokine production was observed in mice immunized with A172-HK and 20 weeks later infected with A170 (data not shown). Given the abundant release of pro-inflammatory cytokines during *S. aureus* infection [36]–[37], this property of A172 is biologically relevant.

### Selection for phage resistance as a general approach for vaccine production

Three groups of mice were immunized (10^8^ CFU/mouse, intramuscularly) with the M^Sa1^-resistant strain A178 (derived from the M^Sa1^-susceptible isolate A177), M^Sa2^-resistant strain A180 (derived from the M^Sa2^-susceptible isolate A179) or M^Sa3^-resistant strain A182 (derived from the M^Sa3^-susceptible isolate A181), respectively. Two weeks later, immunized and control mice were challenged with A170 (10^8^ CFU, intramuscularly). The phage-resistant strains all provided protection in terms of survival and level of host colonization (Table 5; P: 0.01–0.02) and – like A172 - all lost t-GlcNAc (data not shown) while all gained the capacity to secrete capsular polysaccharide (Figure 3). The experiment demonstrates the general applicability of selection for phage resistance as a means to curb bacterial virulence. The experiment demonstrates also how different *S. aureus* isolates use the same defence strategy against different phages.

**Table 5:** Selection for phage-resistance as a general approach for *S. aureus* vaccine production.

Vaccination			Interval vaccination-challenge (weeks)	Challenge					
Dose (CFU/mouse)	Strain	Route		Dose (CFU/mouse)	Strain	Route	Survival	P value^b^	CFU/g^a^ (mean ± SD)
10^8^	A178	Im	20	10^8^	A177	Im	9/10	0.01	230±40^c^
			-	-	-	-	0/10		4.6×10^8^±1.5×10^8^
10^8^	A180	-	20	10^8^	A179	Im	10/10	0.01	160±34^c^
			-	-	-	-	0/10		5.3×10^8^±2×10^8^
10^8^	A182	-	20	10^8^	A181	Im	8/10	0.02	180±25^c^
			-	-	-	-	0/10		3.5×10^8^±1.2×10^8^

a Colony forming units per g of kidneys; value calculated on 3 mice.

b Two tailed Fisher's exact test.

c P<0.001.

### The capsular polysaccharide from A172 provides protection

**Figure 8:**
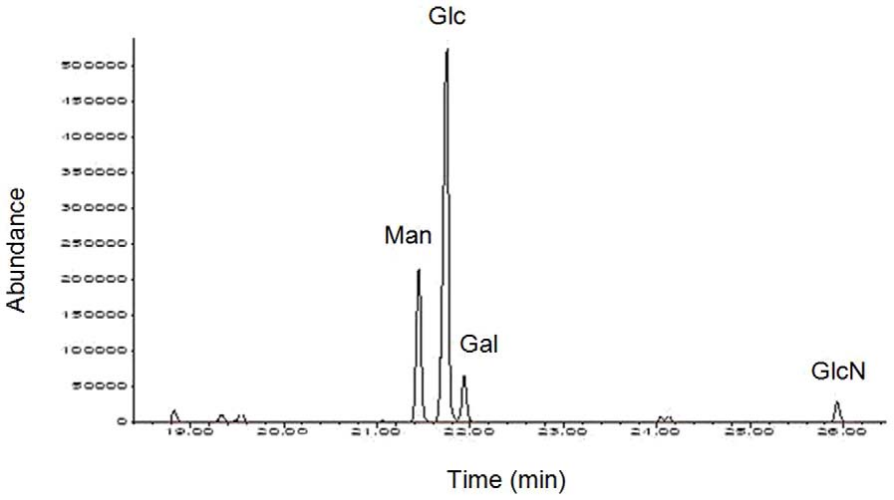
Gas liquid chromatography profile of monosaccharides obtained by acid hydrolysis of the capsular polysaccharide from *S. aureus* A172. Abundance expresses the relative ratio between monosaccharides.

Capsular polysaccharide (CP) production is a characteristic common to the four protective strains (A172, A178, A180, A182) (Figure 3). In an attempt to identify the protective component of A172, the CP from this strain (CP-A172) was purified and analyzed by gas liquid chromatography (GLC). CP-A172 contains glucose and mannose in the molar ratio of 3∶1 and traces (molar ratio: 0.3) of galactose and GlcN (Figure 8). Immunization of mice with CP-A172 (25 µg/mouse) or treatment with the serum from mice immunized with CP-A172 (10 µl diluted 1∶10 with sterile saline/mouse) protected mice against a lethal dose of A170 (P: 0.004; Table 6, experiments 1–2).

**Table 6:** Active and passive protection afforded by CPA172.

	Treatment			Interval treatment challenge	Challenge					
Exp	Treatment	Dose µg/mouse	Route		Dose CFU	Pathogen	Route	Survival	P value^b^	CFU/g^a^ (mean ± SD)
1	CPA172	25	Im^c^	20 weeks	10^8^	A170	Im	20/20	0.004	138±27^d^
					-	-	-	0/10		4×10^8^±10^7^
2	Anti-CPA172	10	Im	24 h	10^8^	A170	Im	20/20	0.004	205±42
	Normal mouse serum	10	-	-	-	-	-	0/10		3.8×10^8^±10^7^

a Colony forming units per g of kidneys; values calculated on 3 mice.

b Two tailed Fisher's exact test.

c Intramuscular.

d P<0.001.

## Discussion

Several virulence factors of *S. aureus* are being investigated for their potential use as vaccines [37]–[38]. However, in view of the numerous virulence factors produced by this pathogen [39], it is unlikely that a single component vaccine might protect against a multitude of virulence factors. Also, virulence genes often display high levels of variability, as result of the selection pressure to evade the immune system of the host [40]. The single antigen may therefore have limited distribution among clinical isolates or may not be expressed in vivo. The objective of the present study was to develop a live attenuated *S aureus* strain containing multiple antigens and therefore likely to elicit cross-protection against a spectrum of pathogenic strains. The attenuated strain (A172) proved effective as vaccine even when heat-killed. Thus, heat-killed A172 (A172-HK) possesses the advantages of a multiple component vaccine, without the problems of safety (reversion to phage sensitivity and the accompanying virulent phenotype), distribution and manufacturing of the live vaccines [41].

Phage-resistance often (though not always) occurs at a fitness cost [6], [7]. This trade-off explains why phage-resistant and phage-sensitive bacteria coexist [42]. Bacterial selection for phage-resistance was therefore exploited as an approach to isolating an attenuated strain of *S. aureus*. The approach had already been used successfully to produce a vaccine against *Salmonella enterica* serovar Paratyphi B [18]. The phage-sensitive strain A170, following growth in vitro in the presence of the M^Sa^ phage, yielded the phage-resistant strain A172. Given their common origin, the two strains share several properties, but display also crucial differences. They share the spa-type (t6668), the MLST type (ST 45), the egc type (egc-4) and part of the genome (Figure 1). At the same time, A170 and A172 differ in their teichoic acid structure and the capacity to secrete CP. The teichoic acids of A172 lack the terminal GlcNAc residues (Figure 6B) that phages often use for adsorption on the cell wall of *S. aureus* [28]. More importantly, GlcNAc and the teichoic acids from A170 (A170^TA^) inhibit lysis by the M^Sa^ phage, while glucose or the teichoic acids from A172 (A172^TA^) do not (Figure 5C). These results suggest that A172 gained phage-resistance by losing the phage adsorption site (terminal GlcNA). However, A172 also displays CP production, a strategy that some bacteria put in action to mask their phage adsorption site [43]. In conclusion, while teichoic acid alteration is the likely mechanism adopted by A172 to gain phage resistance, the contribution of CP production cannot be excluded.

In a mouse model of infection, A172-HK elicited high levels of protection against lethal doses of the A170 strain (P 0.004; Table 1, experiment 3). A single dose of A172-HK, without use of adjuvant, was fully protective after vaccine administration by the intramuscular or aerosol routes and protection lasted at least 20 weeks (Tables 1–3). Significantly, A172-HK was effective against methicillin- resistant (MRSA) or vancomycin-intermediate clinical isolates of *S aureus* (VISA) (Table 2). Sometime cross-protection has been attributed to the persistence of the vaccine strain within the host [44]. This possibility can be excluded in the present study since the vaccine persists in the immunized animals for only 8 days, while mice were challenged with virulent *S. aureus* 20 weeks after vaccine administration (Table 1).

Passive immunization of mice with sera from A172-HK vaccinated mice provided protection against the challenge with a lethal dose of *S. aureus* A170. In two independent experiments, 10/10 of the animals passively immunized with an individual dose of 10 µl immune serum (diluted 1∶10 with sterile saline) survived, while 10/10 untreated control mice died (P: 0.01; Table 4, experiment 1). When the experiment was repeated using a higher dose (20 µl immune serum diluted 1∶10 with sterile saline/mouse), 40%–50% of the animals died (Table 5, experiment 2). These results indicate that high antibody doses can be detrimental. This possibility must be considered when interpreting passive protection experiments.

A172-HK was protective in vivo, while A170-HK was not (Table 1). This suggested that A172, along with the resistance to the M^Sa^ phage, also acquired the capacity to express a new molecule, that acts as a protective antigen. Also, the phage-resistant strains (A172, A178, A180, A182) all display the characteristic of secreting CP (Figure 3). CPs are heat stable and can confer protection [45]–[46]. Taken together, these facts provided sufficient ground to claim that the protective efficacy of A172 could reside in its CP (CP-A172). The results confirmed that CP-A172 provides mice with both active and passive resistance against a lethal dose of A170 (Table 6). The main components of CP-A172 are mannose and glucose (Figure 8). This feature distinguishes CP-A172 from the poly-N-succinyl β-1-6 glucosamine vaccine described by McKenney [45] and the capsular polysaccharide vaccine, that has acetylated fucosamine and mannosamine as major components [46].

When compared with A170 infected mice, A172-HK vaccinated animals displayed an elevated transcription level of IL-6 (Figure 7). IL-6 is an anti-inflammatory cytokine controlling the expression of pro-inflammatory cytokines (IFN-γ, I1-β and TNF-α) [36]–[38]. Staphylococcal infections cause a profound release of proinflammatory cytokines, which enhance endothelial cell permeability and extravasation of bacteria from blood to the tissues [38]. In particular, elevated levels of IFN-γ cause necrosis in the kidneys of *S. aureus*-infected mice [37]. Thus, the A172 vaccine, inducing a balanced pro- and anti-inflammatory response, tempers the detrimental effects of an excessive inflammation.

It is not uncommon for clinical isolates to be resistant to staphylococcal phages. This finding deserves a final comment. Resistant bacteria, despite of their reduced fitness, sometimes persist because they attenuate the impact of phages on sensitive bacteria and thus contribute to maintain diversity within the bacterial population [47].

In conclusion, given the multiple virulence factors expressed by *S. aureus*, a whole cell bacterial vaccine would have significant potential. However, the application of these vaccines (against *S. aureus* and bacterial pathogens in general) is limited by the difficulty of developing reliable procedures of bacterial attenuation [41]. Selection for phage resistance promises to serve this purpose well. Of course, it must be recalled that the trade-off associated with phage resistance can be paid by the pathogen in a form other than lowered virulence. With these caveats, the evidence so far available – [18] and this article - suggests that selection for phage resistance as a means to curb bacterial virulence is not particular to the phage M^Sa^ or the bacterial strain A172, but more general. Phage resistance, long considered a problem in the fight against bacteria [48], is shown here to be an opportunity.
